# Hashim, H. *et al*., Cytoprotective Effect of Benzyl *N'*-(5-Chloro-indol-3-yl-methylidene)hydrazinecarbodithioate against Ethanol-induced Gastric Mucosal Injury in Rats. *Molecules* 2012, *17*, 9306-9320

**DOI:** 10.3390/molecules18077910

**Published:** 2013-07-05

**Authors:** Harita Hashim

**Affiliations:** Department of Biology, Faculty of Applied Science, University Teknologi MARA, Shah Alam 40450, Malaysia

I have been made aware of that fact that substantial parts of our paper published in *Molecules* [1] duplicate the contents of another paper previously published under our names in *African Journal of Pure and Applied Chemistry* [2] of which existence I was unaware. Although the compounds reported in both papers are different, I was not aware of the same biological data being used in the earlier publication. The article in *Molecules* was submitted in good faith based on the collective work that was presented to me by our co-author Dr Mughrabi, listed as the corresponding author of the article published in *African Journal of Pure and Applied Chemistry* [2].

Since becoming aware of this duplication, I have been in contact with our colleague for some explanation as to how different compounds could produce the same data but unfortunately the explanation was not satisfactory. In light of this, we would like to withdraw our paper from *Molecules*. As corresponding author, I take full responsibility for the error in failing to completely check the data presented. I would like to apologize on behalf of my co-authors to the readership of *Molecules* for any inconveniences caused by this error.
